# Dynamic Prediction of Near-Term Overall Survival in Patients with Advanced NSCLC Based on Real-World Data

**DOI:** 10.3390/cancers14030690

**Published:** 2022-01-29

**Authors:** Xuechen Wang, Kathleen Kerrigan, Sonam Puri, Jincheng Shen, Wallace Akerley, Benjamin Haaland

**Affiliations:** 1 Department of Population Health Sciences, Division of Biostatistics, University of Utah, Salt Lake City, UT 84108, USA; jincheng.shen@hsc.utah.edu (J.S.); Ben.Haaland@hsc.utah.edu (B.H.); 2 Department of Internal Medicine, Division of Oncology, Huntsman Cancer Institute, University of Utah, Salt Lake City, UT 84112, USA; Katie.Kerrigan@hci.utah.edu (K.K.); Sonam.Puri@hci.utah.edu (S.P.); wallace.akerley@hci.utah.edu (W.A.)

**Keywords:** time-dependent features, landmarking, dynamic prediction, overall survival, IPCW AUC, model-based calibration

## Abstract

**Simple Summary:**

Patients near the end of life often receive aggressive care, which may be of low value. For patients with advanced cancers, it is standard clinical practice to estimate the prognosis to inform treatment decisions and improve end-of-life care. However, clinical estimates of prognosis may be imprecise and rapidly become out-of-date if clinical factors that evolve over time are not incorporated. Patient prognosis is commonly estimated based on a clinician’s subjective assessment of patient reserve, such as performance status. We propose a spline-smoothed landmarking approach to dynamically estimate survival probabilities based on objective, evolving patient features. The proposed method allows predictions at any time during the patient disease course and demonstrates dramatically improved prediction accuracy compared to methods using clinical features at a fixed time. The proposed approaches can assist clinicians and patients in appropriately regulating treatments to improve outcomes and quality of life.

**Abstract:**

Patients with terminal cancers commonly receive aggressive and sub-optimal treatment near the end of life, which may not be beneficial in terms of duration or quality of life. To improve end-of-life care, it is essential to develop methods that can accurately predict the short-term risk of death. However, most prediction models for patients with cancer are static in the sense that they only use patient features at a fixed time. We proposed a dynamic prediction model (DPM) that can incorporate time-dependent predictors. We apply this method to patients with advanced non-small-cell lung cancer from a real-world database. Inverse probability of censoring weighted AUC with bootstrap inference was used to compare predictions among models. We found that increasing ECOG performance status and decreasing albumin had negative prognostic associations with overall survival (OS). Moreover, the negative prognostic implications strengthened over the patient disease course. DPMs using both time-independent and time-dependent predictors substantially improved short-term prediction accuracy compared to Cox models using only predictors at a fixed time. The proposed model can be broadly applied for prediction based on longitudinal data, including an estimation of the dynamic effects of time-dependent features on OS and updating predictions at any follow-up time.

## 1. Introduction

Lung cancer is the most common cancer worldwide and the third most common cancer in the United States of America (USA) [1,2]. Meanwhile, it is the leading cause of cancer-related death in both men and women in the USA [3,4]. Non-small-cell lung cancer (NSCLC) is the most common type of lung cancer, making up ~84% of all lung cancer diagnoses. Advanced lung cancer usually refers to stage 3B or 4, and ~58% of NSCLC patients are already advanced when they are diagnosed [5]. Generally, advanced lung cancer will develop into a progressive terminal disease, with a 5-year survival rate of under 7% [6]. For patients with advanced or metastatic cancer, it is standard clinical practice to estimate the prognosis as a means to assess tolerance to therapy and the usefulness of treatment. Several studies have shown an increasing trend in continuing aggressive care for patients near the end of life, which is costly and may be of low value [7,8]. It is essential to develop methods that can accurately recognize when a patient is approaching the end of life.

In most studies, the proposed survival prediction models for patients with lung cancer are static prediction models [9,10,11]. Traditional static prediction models take available patient features at a fixed time, commonly the time of diagnosis or initiation of therapy. At a time when well-structured electronic databases were not commonly available, these static predictions were often the best prognostic tools available. However, with the development of technology, substantial longitudinal clinical information on patients has become available in electronic health records, which has highlighted the limitations of static prediction models. First, static prediction models are not able to take advantage of longitudinal measures that reflect evolving patient features. Intuitively, it may be beneficial to incorporate the most recent clinical features, such as lab values, biomarkers, or measures of patient reserve, such as performance status, into estimates of a patient’s present prognosis. Second, static survival prediction models, which only use baseline information, cannot account for the changing at-risk patient population. While a static model may be suitable for patients at baseline, its application to a patient who has lived 6 months after baseline may lead to a biased prediction. A static model may not capture the varying association between clinical factors and outcomes over patient disease course. Here, we propose a dynamic prediction modeling approach that can address the above issues.

Landmarking is a dynamic prediction approach, as described by Van Houwelingen [12]. The fundamental idea of landmarking is to adaptively construct a model using a collection of patients who are still at risk at each corresponding time point [13]. For the survival prediction of patients with advanced NSCLC, besides fixed patient demographics, longitudinal factors and medication history are potential predictors. The landmarking approach allows the inclusion of a relatively large number of time-dependent predictors in the model without introducing excessive computation. Further, a modeling strategy based on the cohort of at-risk patients is transparent and is intuitively appealing to physicians and researchers without extensive training in statistics. Joint modeling is another widely used approach to dynamic prediction. However, joint modeling requires the complete specification of models for longitudinal factors, a model for the survival outcome, and a method to link them, which presents concerns about model mis-specification. Importantly, it is commonly infeasible to correctly specify models for all of the time-dependent predictors simultaneously. Further, survival prediction using the joint modeling method often requires numeric integration and substantial computation. Meanwhile, it has been shown that the computation for fitting joint models with only a few time-dependent predictors may be infeasible or unstable, especially when the available sample is small and the longitudinal predictors are measured sparsely [14]. In order to avoid imposing restrictive models for time-dependent predictors and link the functions that need comprehensive subject knowledge, we chose to adopt the landmarking approach. 

Our research was motivated by the end-of-life care problem for patients with advanced NSCLC and was based on real-world electronic health record (EHR) data. Numerous time-dependent clinical factors were collected, including performance status and lab values. Performance status is routinely used in clinical practice to evaluate how a patient’s disease is progressing and affecting their daily living, determine treatment, and estimate their prognosis [15]. However, performance status depends on the clinician’s subjective assessment of the patient. One study illustrated that considerable variability exists in Eastern Clinical Oncology Group (ECOG) performance status (PS) determined by clinicians [16]. Ideally, prognostic tools would be based on objective clinical factors, for example, lab values or activity trackers. Unlike patient demographics such as date of birth and sex, clinical factors that are measured at multiple time points may provide a window on evolving patient status. We propose a spline-smoothed dynamic prediction model using the landmarking approach, which has smoothly varying landmark-dependent associations and is easy to implement. In actual practice, clinical visits are not lined up for each patient, which implies irregularly spaced measurements of time-dependent clinical factors. The traditional landmarking approach does not account for these irregularly spaced measurements. The proposed method is able to deal with irregular and non-aligned measurements with no extra effort.

The proposed model can be widely applied for prediction based on longitudinal data, including an estimation of the effects of time-dependent covariates on overall survival and updating the survival prediction at any follow-up time with newly available information. The proposed model may help clinicians provide accurate and objective estimates of patient prognosis as well as inform treatment and care decisions. The remainder of this paper is organized as follows. In Section 2, we formally describe the proposed approach as well as measures of discrimination and calibration to assess predictive quality in the context of dynamic prediction for survival outcomes. The proposed dynamic prediction model is applied to longitudinal data to objectively estimate prognosis for patients with advanced NSCLC from the USA Flatiron Health nationwide electronic health record-derived database. Results and predictive performance comparisons are summarized in Section 3, with the discussion and conclusions in Section 4 and Section 5.

## 2. Materials and Methods

### 2.1. Data

Analyses were based on data from the USA Flatiron Health nationwide electronic health record-derived, de-identified database comprising patient-level structured and unstructured data curated via technology-enabled abstraction [17,18]. Overall survival analyses were based on a composite mortality variable that aggregates EHR-derived structured and unstructured information, as well as third-party death surveillance sources. At the time we started the study, the de-identified data originated from approximately 280 USA cancer clinics (~800 sites of care) [19]. The study was conducted according to the guidelines of the Declaration of Helsinki and approved by the Institutional Review Board of the University of Utah, which includes a waiver of informed consent.

The study consisted of observations on patients receiving at least one line of treatment for advanced NSCLC with advanced diagnosis dates from 1 January 2011 to 1 June 2019, seen by 129 providers, at 127 community practices and two academic medical centers in the USA. In addition, patients were restricted to those who had a visit or medication order within 90 days of advanced diagnosis to minimize the potential impacts of patients who were not primarily engaged with the relevant practice, for example, patients seeking a second opinion. Patient data were collected through 1 June 2019, which provided at least 6 months of potential follow-up for all patients. Overall survival was from the initiation of first-line therapy to the date of death and was censored at the last visit date or end of most recent oral therapy. Our analyses considered a set of seven covariates assessed at or before the initiation of first-line therapy (baseline hereafter): age, gender, smoking history, targetable mutation status, race and ethnicity, histology, and first-line treatment; a set of baseline biomarkers, lymphocyte counts and weight, which were taken from up to 30 days prior to baseline until baseline; and two time-dependent predictors, albumin and ECOG PS, which were collected at clinical visits. Patients who did not have any measurements of the time-dependent predictors were excluded from the analyses.

### 2.2. Landmark Approach

**Figure A1:**
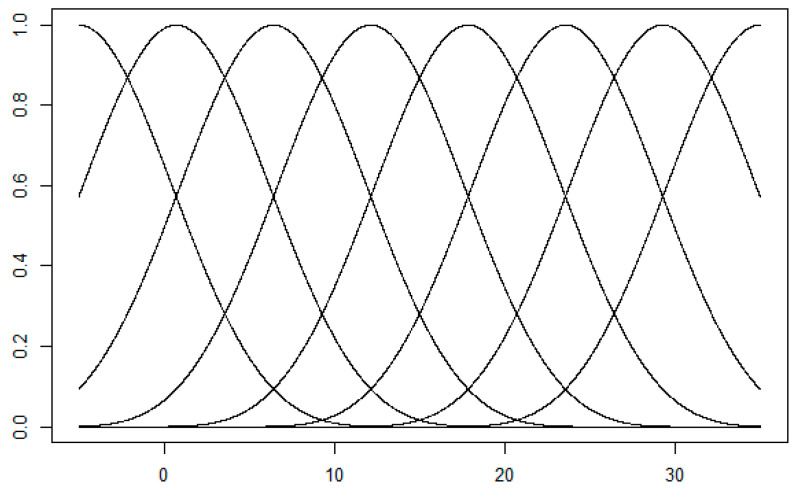
Distribution of spline basis functions based on landmarks.

In this study, we propose a spline-based Cox proportional hazards (PH) model along with a landmarking approach that allows the incorporation of time-dependent covariates and estimates dynamic effects over time [20]. The proposed method uses all patients remaining at risk at the landmark time for prediction and to estimate the parameters. A landmark represents a time point in the disease course (on or after baseline) at which an estimate of future patient prognosis is desired. For a single landmark, s, the postulated model is:

(1) $$h_{s}\left(t|X\left(s\right),Z\right)=h_{s,0}\left(t\right)exp\left(X\prime \left(s\right)\beta_{s}+Z^{\prime }\theta \right),\;for\;t\geq s.$$

where, s is the landmark time of prediction and t (>s) is a future time of interest also known as the horizon time. X(s) denotes the vector of (potentially) dynamic covariates at landmark s, and $\beta_{s}$ is the vector of parameters (log hazard ratios) at landmark s. Z denotes the vector of time-fixed covariates, and θ is the corresponding vector of parameters. $h_{s,0}\left(t\right)$ is the baseline hazard rate for landmark s, and $h_{s}(t|X\left(s\right),\;Z)$ is the hazard rate with particular covariates (X(s) and Z) at time t after s. In order to obtain smoothed time-varying parameters, we propose a spline-based landmark model in which $\beta_{s}$ can be expanded as:

(2) $$\beta_{s}=\overset{k}{\underset{i=1}{\sum }}\alpha_{i}\varphi_{i}\left(s\right),\;$$

where k is the number of spline basis functions, and $\varphi_{i}\left(s\right)$ represents the $i^{th}$ basis function [21,22]. The number of basis functions is determined according to the distribution of landmarks of interest (Figure A1). Note that the baseline hazard rate also depends on the landmark in this model. Here, we estimate baseline hazard rates separately for each integer month landmark, which is a clinically meaningful time interval. The choice of when to re-estimate baseline hazard rates should be adapted to the specific context of individual studies. Clearly, a static prediction model is a special case of the DPM when there is only one particular landmark time of interest (i.e., baseline).

While landmarks can be any time at which prediction of survival is needed, we considered landmarks of interest as integer months after baseline, aligned with the time unit for the re-estimation of baseline hazard rates. For a specific landmark, the presently available predictors and history would be appropriate for use in the dynamic prediction model. When there are multiple measurements for a predictor within a particular month of interest, the most recent value for that month was used for prediction. We generated a longitudinal dataset in which each measurement time was converted into months after baseline (i.e., the landmark). We used this dataset to fit spline-based landmark models with robust standard errors clustered by patient to account for repeated measurements within each patient [23].

### 2.3. Prediction Accuracy Assessment

Time-to-event outcomes are common in medical applications, and risk prediction (prognosis) is of great interest to clinicians and researchers. Assessing the performance of a prediction model is essential. Two key elements of predictive model assessment are model discrimination and model calibration [24].

#### 2.3.1. Discrimination

Discrimination characterizes the model’s ability to accurately rank subjects’ risk of events from low to high. In the analyses for binary outcomes, a frequently utilized model discrimination statistic is the area under the receiver operating characteristic (ROC) curve (AUC), or equivalently (for binary outcomes), the concordance statistic (C-statistic) [24]. An ROC curve plots the sensitivity against 1-specificity for all possible cutoffs, which can separate subjects as having a predicted outcome or not using the predicted probabilities [25]. The C-statistic is the proportion of subject pairs that have agreement on the order of predicted survival probabilities and observed time-to-event lengths among all ordered pairs [26]. Various extensions of C-statistic and AUC are available in the context of right-censored time-to-event outcomes [27,28]. Here, an inverse probability of censoring weighted (IPCW) AUC proposed by Hung and Chiang is used to estimate AUC at fixed time horizons [29]. The goal of IPCW is to correct the selection bias caused by censoring in time-to-event outcomes. The observations on uncensored subjects at a particular time are weighted via the conditional probability of being uncensored. The time-dependent AUC and C-statistic respectively provide a summary of accuracy at a specified time and an overall measure of predictive accuracy. In this study, longitudinal lab values were considered in the prediction models and lab values would commonly change over time, which may limit their value for long-term prediction. Here, we were interested in the prediction of near-term patient outcomes; therefore, AUC for predicting events over a short, fixed time horizon was opted for, instead of the C-statistic that examines concordance across all observed horizon times.

The data were divided into training and validation sets. Two-thirds of the patients were randomly selected into the training set, and the remaining patients were in the validation set. Models were developed in the training set while the validation set was used for model assessment. A Cox PH model was utilized to estimate the probability of censoring conditional on age and gender. In order to be able to assess the landmarking DPM, we proposed the following time-dependent IPCW AUC. As near-term events are what we were most interested in, model performance was assessed for predicting patient events in the future at horizons of half a month, 1 month, 3 months, and 6 months across landmark times of 0 through 12 months from first-line initiation.

For patient i, let $T_{i}$ denote the true, potentially unobserved time-to-event (overall survival, here), $C_{i}$ the censoring time, $\delta_{i}=I\left(T_{i}\leq C_{i}\right)$ the indicator of death, ${\tilde{T}}_{i}=\min\left(T_{i},\;C_{i}\right)$ the observed time, X(s) time-dependent covariates at landmark time s, Z time-fixed covariates, and $n_{s}$ the number of patients at risk at landmark s. Let ${\hat{S}}_{s}\left(t|X\left(s\right),\;Z\right)$ denote the estimated survival probability of living beyond t into the future from landmark time s conditional on predictors X(s) and Z. Let ${\hat{S}}_{s,C}\left(t\right)=P(C_{i}>t|X_{i}\left(s\right),\;Z,\;{\tilde{T}}_{i}>s)$ denote the estimated censoring probability at the horizon time t from the landmark time s. Further, let u denote a possible cutoff for flagging a patient as having a predicted event on or before time t after landmark time s. Then, the IPCW sensitivity and specificity are:

(3) $$\hat{Se_{s}}\left(u,t\right)=\frac{\sum_{i=1}^{n_{s}}I\{{\hat{S}}_{s}\left(t|X_{i}\left(s\right),\;Z,\;{\tilde{T}}_{i}>s\right)<u,\;{\tilde{T}}_{i}\leq s+t\}\frac{\delta_{i}}{n_{s}{\hat{S}}_{s,C}\left({\tilde{T}}_{i}\right)}}{\sum_{i=1}^{n_{s}}I\left\{{\tilde{T}}_{i}\leq s+t\right\}\frac{\delta_{i}}{n_{s}{\hat{S}}_{s,C}\left({\tilde{T}}_{i}\right)}}\;,\;{\hat{Sp}}_{s}\left(u,t\right)=\frac{\sum_{i=1}^{n_{s}}I\{{\hat{S}}_{s}\left(t|X_{i}\left(s\right),\;Z,\;{\tilde{T}}_{i}>s\right)\geq u,\;{\tilde{T}}_{i}>s+t\}\frac{1}{n_{s}{\hat{S}}_{s,C}\left(t\right)}}{\sum_{i=1}^{n_{s}}I\{{\tilde{T}}_{i}>s+t\}\frac{1}{n_{s}{\hat{S}}_{s,C}\left(t\right)}}$$

The area under (ROC) ^(t) curve is then:

(4) $$\hat{AUC}\left(t\right)\;=\frac{\sum_{i=1}^{n_{s}}\sum_{j=1}^{n_{s}}I\{{\hat{S}}_{s}(t|X_{i}(s),Z,{\tilde{T}}_{i}>s)<{\hat{S}}_{s}(t|X_{j}(s),Z,{\tilde{T}}_{j}>s)\}*I\{{\tilde{T}}_{i}\leq t,{\tilde{T}}_{j}>t\}\frac{\delta_{i}}{n_{s}^{2}{\hat{S}}_{s,C}\left({\tilde{T}}_{i}\right){\hat{S}}_{s,C}(t)}}{\left(\sum_{i=1}^{n_{s}}I\left\{{\tilde{T}}_{i}\leq t\right\}\frac{\delta_{i}}{n_{s}{\hat{S}}_{s,C}\left({\tilde{T}}_{i}\right)}\right)\left(\sum_{j=1}^{n_{s}}I\{{\tilde{T}}_{j}>t\}\frac{1}{n_{s}{\hat{S}}_{s,C}(t)}\right)}.$$

Ties may occur in real-word data, therefore the term $I\left\{{\hat{S}}_{s}\left(t|X_{i}\left(s\right),\;Z,\;{\tilde{T}}_{i}>s\right)<{\hat{S}}_{s}\left(t|X_{j}\left(s\right),\;Z,\;{\tilde{T}}_{j}>s\right)\right\}$ above can be replaced by $I\left\{{\hat{S}}_{s}\left(t|X_{i}\left(s\right),\;Z,\;{\tilde{T}}_{i}>s\right)<{\hat{S}}_{s}\left(t|X_{j}\left(s\right),\;Z,\;{\tilde{T}}_{j}>s\right)\right\}+\frac{1}{2}I\left\{{\hat{S}}_{s}\left(t|X_{i}\left(s\right),\;Z,\;{\tilde{T}}_{i}>s\right)={\hat{S}}_{s}\left(t|X_{j}\left(s\right),\;Z,\;{\tilde{T}}_{j}>s\right)\right\}$ to account for any ties.

#### 2.3.2. Calibration

Model calibration is another aspect of the quality of a prediction model. Calibration refers to how accurately the model’s predictions match actual event rates. The Hosmer–Lemeshow (HL) goodness-of-fit test is a well-known method for a calibration assessment in the binary setting. It is commonly computed by dividing the study population into 10 groups using deciles of the predicted risk scores based on the predictive model whose calibration is being assessed [30]. A model-based approach proposed by Crowson et al. is asymptotically equivalent to the HL test for binary outcomes and views the calibration in a regression context. The model calibration process is more complex for a time-to-event prognostic model given the spectrum of at-risk times across patients. One important advantage of the model-based approach is the natural extension to Cox models for time-to-event outcomes [31]. In this study, a method that also requires separating the study population into groups was applied.

As the proposed DPM is built on a Cox model, the martingale residuals at landmark s can be computed by $m_{i,s}=\delta_{i}-e_{i,s}$ for each subject i, where $\delta_{i}$ is the indicator of death for subject i and $e_{i,s}$ represents the expected number of events estimated at landmark s [32]. In detail, $e_{i,s}=H_{s,0}\left(t\right)exp\left(X{\left(s\right)}^{\prime }\beta_{s}+Z^{\prime }\theta \right)$, where $H_{s,0}\left(t\right)$ is the baseline cumulative hazard at landmark s. The martingale residuals can be interpreted as the difference between the observed number of events and the expected number of events under the Cox model. In other words, the martingale residuals estimate the number of observed events that are not predicted by the model [33]. A good prediction model tends to have smaller absolute martingale residuals. The quantity that is required for validation at landmark s for subject i in the model-based calibration method is $log\left(e_{i,s}\right)$. In a survival analysis setting, Poisson regression is the appropriate method for a model-based assessment of calibration. It is well known that a Poisson regression with pre-specified hazard rates within specified time intervals is equivalent to a Cox model [34]. The reason for not using a Cox model in calibration is that it would confound the evaluation of absolute risk by calculating a new baseline hazard. When using the model-based calibration approach, one challenge of using the predicted number of events to build groups is the incorporation of the follow-up time, which may vary across patients. Instead, predicted survival probabilities at a fixed horizon time of 1 month were employed to build groups in our study. In a well-calibrated model, the observed and expected number of events should agree up to sampling variability across groups of patients.

The model-based endpoint for calibration can be expressed as:

(5) $$E\left(\delta_{i}\right)=exp\left\{\gamma_{1,\;s}*group_{1}+\ldots +\gamma_{q,s}*group_{q}+log\left(e_{i,s}\right)\right\}$$

where q is the number of risk strata groups, commonly 10 risk groups for the HL test. As described above, groups are identified using the survival probabilities at a fixed horizon time. The coefficient before the expected number of events ($e_{i,s}$) is forced to be 1, and no intercept is estimated in the model. Then, if any of the coefficients of the groups (any of the γs) has a statistically significant difference from 0, it means the predicted number of events in that group is not well aligned with the observed number of events.

### 2.4. Bootstrap

Patient-wise bootstrapping with 100 iterations was applied to construct 95% confidence intervals for the IPCW AUC at half-month, 1-month, 3-month, and 6-month horizons in the patient’s future from disease course landmarks of 0 through 12 months from first-line initiation. Prediction models were built using the training data. Within each bootstrap iteration, a random sample with a replacement of patients was obtained based on the validation dataset, and the within-bootstrap IPCW AUC was estimated using this sample [35].

### 2.5. Model Building

In order to compare the predictive performance of a spectrum of dynamic prediction models, a few variations to predictive modeling were considered. The first approach utilizes a Cox PH model with fixed covariates including age, gender, smoking history, targetable mutation status, race/ethnicity, histology, first-line treatment, baseline weight, ECOG PS, lymphocyte counts, and albumin. The second approach utilizes a dynamic prediction model with time-dependent ECOG PS and/or albumin. The third approach utilizes a dynamic prediction model with the time-dependent covariates as well as the baseline covariates (those that were included in the first modeling approach). The first approach is a static multivariable prediction model, the second approach is a dynamic prediction model without fixed covariates, and the third approach is a dynamic prediction model with baseline covariates. Cox PH models stratified by the landmarks provide separate baseline hazard rates at different landmarks. The proposed DPM allows the effect of time-dependent covariates to vary smoothly across landmarks.

## 3. Results

### 3.1. Patients, Baseline Characteristics, and Summary of Overall Survival (OS)

**Figure 1:**
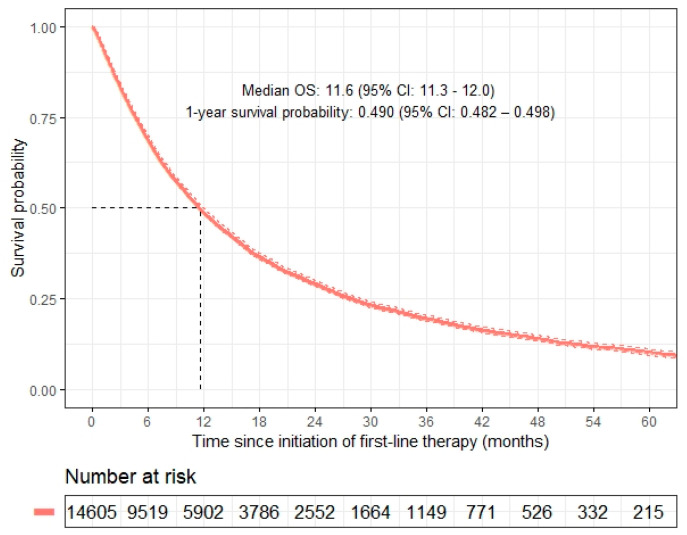
Kaplan-Meier estimates of overall survival for the advanced NSCLC patients.

**Figure A2:**
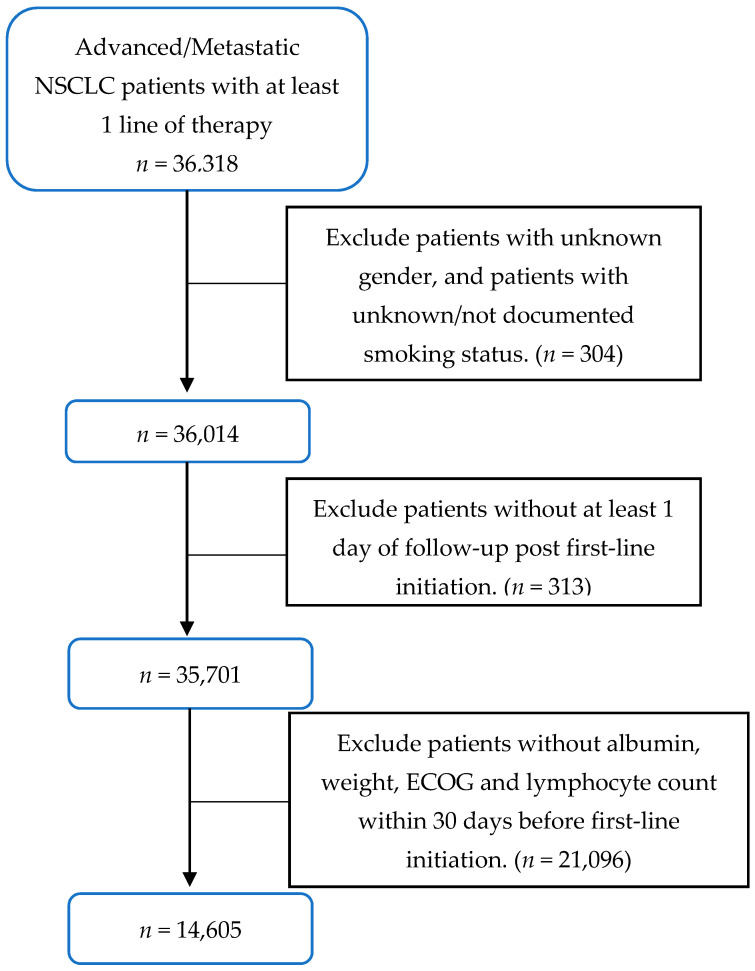
Flowchart of the data cleaning approach.

The procedure for identifying the analytic cohort is depicted in a flowchart in Appendix A (Figure A2). In the Flatiron Healthcare database, 36,318 advanced NSCLC patients were selected, who also met the criteria outlined in Section 2. Patients who did not have information on the factors that were included in the Cox model using baseline covariates and did not have at least one day of follow-up after baseline were excluded. The primary analysis included 14,605 patients. The patient characteristics at baseline are summarized in Table 1. The mean age at baseline was 68.2 years; 46.3% (*n* = 6769) of patients were female; 89.5% (*n* = 13,071) had a history of smoking; 71.9% (*n* = 10,498) were non-Hispanic white; and 67.9% (*n* = 9917) had non-squamous cell carcinoma histology. Targetable mutations were reported in 8.4% (*n* = 1228) of the study population. ECOG PS at baseline was <2 for 79.2% of the cohort (*n* = 11,563). In total, 50.9% (*n* = 7436) of patients received first-line chemotherapy, 13.7% (*n* = 1999) received first-line immunotherapy, 7.1% (*n* = 1031) received first-line tyrosine kinase inhibitors, and 3.2% (*n* = 473) received study drugs as their first-line therapy. The median overall survival for our study population was 11.6 months (95% confidence interval (CI): 11.3–12.0), the 6-month survival probability was 0.689 (95% CI: 0.682–0.697), and the 1-year survival probability was 0.490 (95% CI: 0.482–0.498) (Figure 1).

### 3.2. Dynamic Prediction Models Considering Only One Time-Dependent Variable

**Figure 2:**
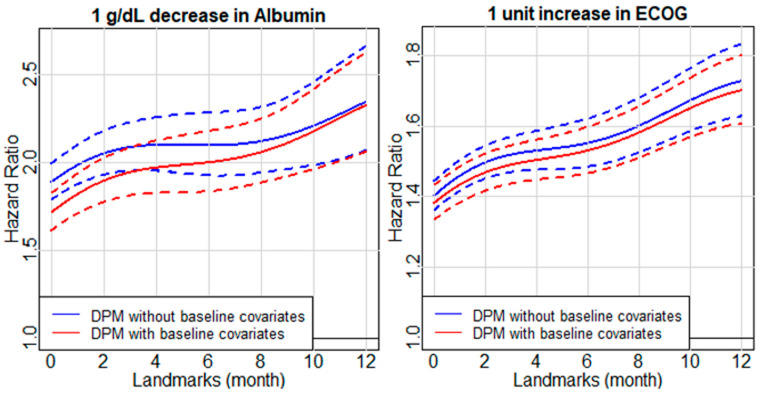
Dynamic hazard ratios for 1 g/dL decrease in albumin (**left**) and 1 unit increase in ECOG PS (**right**) on overall survival. Solid lines represent the dynamic hazard ratios over time and dashed lines represent the corresponding 95% confidence intervals.

Considering the number of patients at risk at each landmark and the number of events occurring afterward, the dynamic effects of each time-dependent covariate were estimated from baseline to 12 months after baseline. A decrease of 1 g/dL in albumin (in serum or plasma) was a negative prognostic feature associated with OS, and the harmful association became stronger as more time passed from baseline (Figure 2). For example, a decrease in albumin of 1 g/dL at baseline, with all other covariates constant, was associated with an increase in the hazard of death by 72% (hazard ratio (HR) = 1.72, 95% CI: 1.61–1.83). At 12 months after baseline, a decrease in albumin of 1 g/dL, with all other covariates constant, was associated with an increase in the hazard of death by 133% (HR = 2.33, 95% CI: 2.06–2.62). We compared the DPM including baseline covariates with a model specifying a constant HR for albumin (i.e., time-varying albumin but constant HR for albumin decrease) across time using a Wald test and found evidence that the model with time-varying albumin and time-varying HR for albumin decrease was a better fit for the data (*p* < 0.001). This suggests that the prognostic impact of albumin strengthens over time. As the patient time passes from baseline, the dynamic HR for decrease in albumin from the DPM with only time-dependent albumin (second approach), converges to that from the DPM with both time-dependent albumin and baseline covariates (third approach), suggesting that the confounding effects of baseline covariates may gradually attenuate.

Time-dependent ECOG PS had a negative prognostic association with OS, which strengthened over time, similar to albumin (Figure 2). An increase in ECOG PS of 1 unit, with all other covariates constant, was associated with an increase in the hazard of death by 38% (HR = 1.38, 95% CI: 1.33–1.43) at baseline, and with a 70% increase in the hazard of death (HR = 1.70, 95% CI: 1.61–1.80) at 12 months after baseline. Note that the ECOG PS scores used in modeling ranged from 0 to 4, therefore HRs for increasing/decreasing ECOG PS only applied within that range. A similar Wald test for time-varying association for albumin was applied to ECOG PS, and the result suggested that the prognostic impact of ECOG PS may strengthen across the disease course (*p* < 0.001).

**Figure 3:**
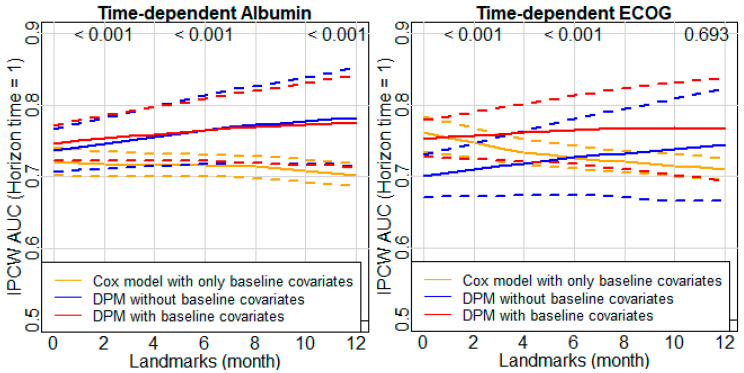
IPCW AUC and 95% CIs for survival prediction at horizon time 1 month from models using time-dependent albumin (**left**), and ECOG PS (**right**). *p* values at the top of each plot are for comparing the DPM using both the time-dependent variable and baseline covariates and the baseline Cox model at landmarks of 2, 6, and 12 months.

**Figure A3:**
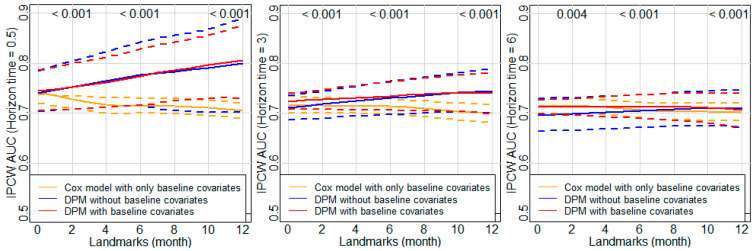
IPCW AUC and its 95% CI for the DPM using time-dependent albumin assessed at horizon time of 0.5 month (**left**), 3 months (**middle**), and 6 months (**right**). *P* values at the top of each plot are for comparing the DPM using both time-dependent albumin and baseline covariates and the baseline Cox model at landmarks of 2, 6, and 12 months.

**Figure A4:**
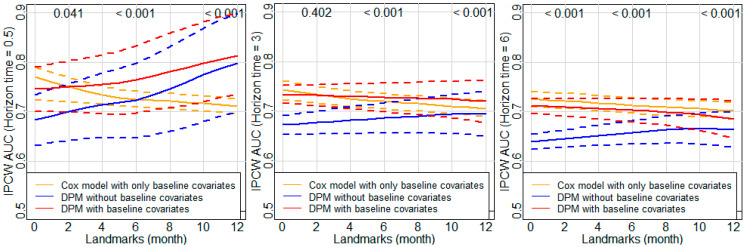
IPCW AUC and its 95% CI for the DPM using time-dependent ECOG PS assessed at horizon time of 0.5 month (**left**), 3 months (**middle**), and 6 months (**right**). P values at the top of each plot are for comparing the DPM using both time-dependent ECOG PS and baseline covariates and the baseline Cox model at landmarks of 2, 6, and 12 months.

**Figure A5:**
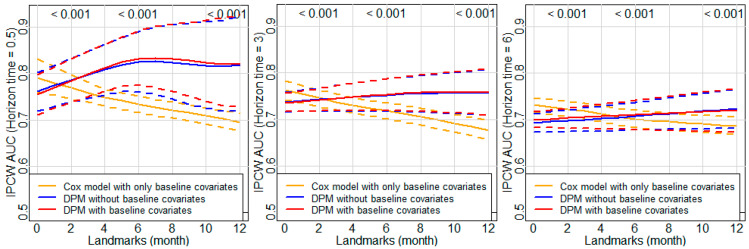
IPCW AUC and its 95% CI for the DPM using time-dependent albumin and ECOG PS assessed at horizon time of 0.5 month (**left**), 3 months (**middle**), and 6 months (**right**). P values at the top of each plot are for comparing the DPM using both time-dependent albumin, ECOG PS, and baseline covariates and the baseline Cox model at landmarks of 2, 6, and 12 months.

In order to make the IPCW AUCs from different models comparable, when considering a particular time-dependent variable, the three candidate models were built based on the same patients who were available at baseline. Moreover, the predicted survival probabilities were estimated at the same future times after baseline. For example, if the DPM is evaluated at a horizon time of 1 month for the landmarks of 1, 2, …, 12, then the baseline Cox model should be evaluated at the corresponding horizon times of 2, 3, …, 13 months after baseline. The IPCW AUCs for DPMs evaluated at a horizon time of 1 month are shown in Figure 3. This figure indicates that the discriminative quality based on the model using a time-dependent variable is better than the model using only baseline covariates. Specifically, the IPCW AUCs for a horizon time of 1 month from both DPMs using time-dependent albumin (second and third approaches) were higher than those from the baseline Cox model (first approach). The differences in AUCs were small for the three models early in the course of disease; then, the differences expanded between the DPMs and the baseline Cox model later in the course of disease. Meanwhile, AUCs for the two DPMs were almost the same over the disease course. Bootstrapping was used to obtain 95% CIs on the AUCs and to compare AUCs among models. AUCs from the DPM including both time-dependent albumin and baseline covariates (third approach) were statistically significantly better than that from the baseline Cox model (first approach) after a landmark of 1 month. Notably, compared to the AUCs from the baseline Cox model, the pattern of AUCs from DPMs using time-dependent ECOG PS was different from what was observed in the DPMs using time-dependent albumin. In particular, AUCs from the DPM using only time-dependent ECOG PS (second approach) were lower than those from the baseline Cox model (first approach) when the landmarks were prior to 6 months from baseline and were higher when the landmarks were after 6 months from baseline. AUCs from the DPM using both time-dependent ECOG PS and baseline covariates (third approach) were almost always higher than those from the baseline Cox model, and the improvements were statistically significant for almost all landmarks. Moreover, the IPCW AUC from the DPM with only time-dependent ECOG PS (second approach) converged toward that from the DPM with both time-dependent ECOG PS and baseline covariates (third approach) over the disease course. Comparisons between time-dependent albumin and ECOG PS suggest that albumin may be of similar strength, or perhaps even more discriminating, regarding the prognostic factor to ECOG PS. The IPCW AUCs for DPMs evaluated at horizon times of 0.5, 3, and 6 months are shown in the Appendix A (Figure A3, Figure A4 and Figure A5).

Comparing the IPCW AUCs at horizon times of 0.5, 1, 3, and 6 months, we found that the predictive performance declined with an increasing time horizon across all candidate models. Furthermore, the AUC measured at horizon times of 0.5 and 1 month for DPMs was substantially higher than that for the baseline Cox model, while the AUCs measured at horizon times of 3 and 6 months for DPMs were similar to or even worse than that for baseline Cox model. These findings suggest that time-dependent albumin and ECOG PS may be able to considerably improve short-term prediction but may be less relevant for longer-term prognosis.

**Figure 4:**
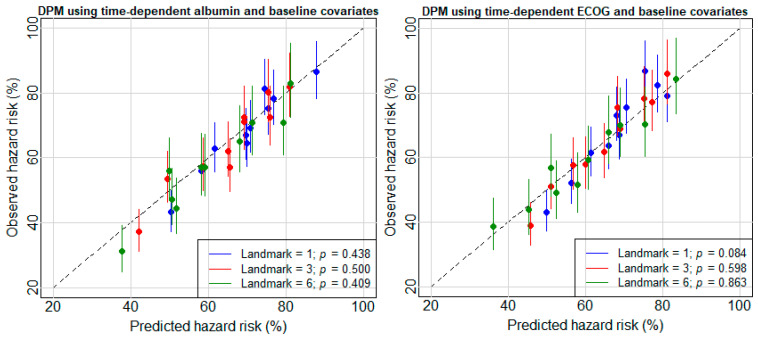
Calibration plot comparing observed and predicted deaths from DPMs using time-dependent albumin (**left**), ECOG PS (**right**), and baseline covariates for patients with advanced NSCLC at landmarks of 1, 3, and 6. Observed hazards for each group of patients and confidence intervals were estimated from a Poisson regression model. The dashed line is the identity line. *p* values are from the Wald test wherein all the coefficients of a group are 0 or not.

Based on IPCW AUC, the DPM with both baseline and time-dependent covariates (third approach) is the most favored model across the scenarios. Therefore, model calibration was assessed for the DPM with both time-dependent variables and baseline covariates at landmarks of 1, 3, and 6 months. Patients in the validation set who were at risk at a particular landmark were partitioned into 10 groups on the basis of the predicted survival probabilities at a horizon time of 1 month. The 95% CIs of the coefficients for all the groups at each landmark crossed the diagonal identity line, suggesting that the DPMs perform well in terms of calibration (Figure 4). In addition, a Wald test was applied to examine whether there existed a group in which the observed number of events was statistically different from the predicted number of events. *p* values for the DPM using time-dependent albumin and baseline covariates at landmarks of 1, 3, and 6 months were 0.438, 0.500, and 0.409, respectively, and *p* values for the DPM using time-dependent ECOG PS and baseline covariates at landmarks of 1, 3, and 6 months were 0.084, 0.598, and 0.863, confirming what is observed in Figure 4.

### 3.3. Dynamic Prediction Model Considering Multiple Time-Dependent Variables

**Figure 5:**
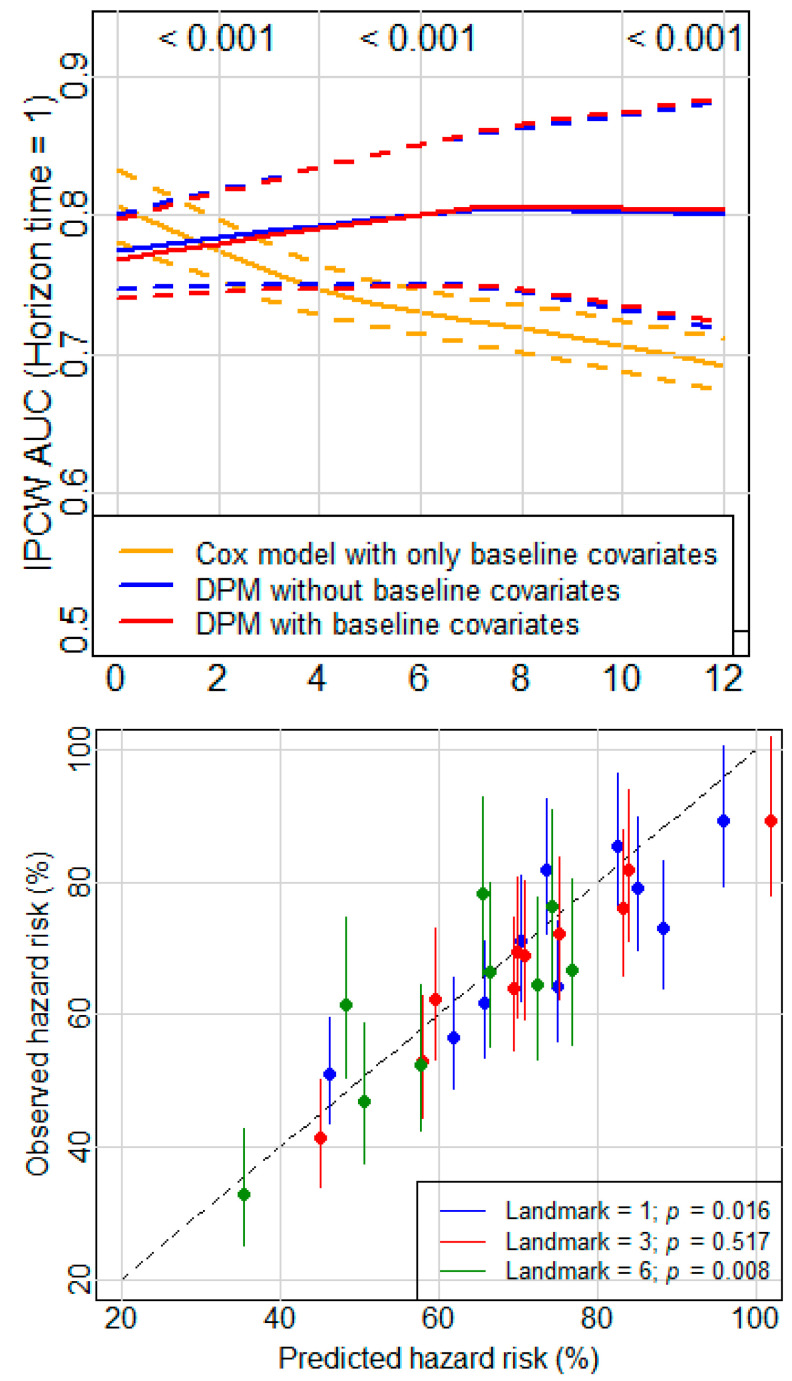
IPCW AUC and calibration plot for the DPM with time-dependent albumin and ECOG PS. *p* values at the top of the left plot are for comparing the DPM using both time-dependent variables and baseline covariates and the baseline Cox model at landmarks of 2, 6, and 12 months. *p* values in the right plot are from the Wald test wherein all the coefficients of a group are 0 or not.

The proposed DPM allows the incorporation of more than one time-dependent predictor. It is conceivable to acquire a more accurate DPM with more available information. A DPM was fit using patients with both time-dependent albumin and ECOG PS, and the number of patients with available data at baseline was reduced by more than 10% compared to the numbers of patients used above. The dynamic hazard ratios for albumin decrease and ECOG PS increase were similar to those displayed in the DPM with only one time-dependent predictor. Importantly, the time-dependent IPCW AUC for this DPM was slightly higher than that for the DPM with only time-dependent albumin and much better than that for the DPM with time-dependent ECOG PS over the disease course. Specifically, the IPCW AUC for the DPM with both time-dependent albumin and ECOG PS was statistically significantly higher than that for the DPM with only time-dependent albumin at landmarks of 0 through 7. The IPCW AUCs for the DPMs after a landmark of 3 months were statistically significantly higher than that for the baseline Cox model. In the calibration plot, a few of the 95% CIs did not cover the identity line, and *p* values of the Wald test at landmarks 1 and 6 were less than 0.05, which suggests that the model incorporating both time-varying albumin and ECOG PS may have been slightly mis-calibrated (Figure 5).

## 4. Discussion

The ultimate goal of this study is to improve end-of-life care by providing an accurate prognosis based on a patient’s evolving clinical factors. We have presented a framework for constructing spline-based dynamic prediction models using a landmarking approach and time-dependent methods for assessing model performance. Near-term prediction using a DPM was significantly improved compared to a static model. The developed DPM can be applied to other longitudinal studies, especially to data with irregularly spaced measurements. In a previous study, a kernel-based DPM using landmarking was proposed that can also deal with irregular measurements [36]. Compared with the kernel approach, the approach proposed in this study may be easier to implement and can use existing statistical software. Additionally, when working with a large real-world dataset and multiple time-dependent predictors, our model is less computationally demanding. Importantly, the DPM can be used to predict future survival not only for new patients at baseline but also for patients at follow-up visits.

The results illustrate that, particularly later in the disease course, the prediction accuracy of a DPM using time-dependent albumin is comparable to, and perhaps even better than, that of a DPM using time-dependent ECOG PS. It may be possible to further improve prediction accuracy by incorporating other time-dependent clinical factors in the DPM. Moreover, it is ideal to obtain a DPM with only objective clinical factors which performs similarly to or even better than a DPM including ECOG PS. The study was restricted by the limited collection of other appealing biomarkers (e.g., absolute lymphocyte count, neutrophil count) and clinical factors. For example, the use of noninvasive ventilation or supplemental oxygen might also be important to support end-of-life care and palliate discomfort for patients with advanced NSCLC [37]. When data on enough patients are available, it may be valuable to build a DPM incorporating all available information. A DPM may provide clinically valuable evidence for physicians to assist their routine practice. With the increasing applications and development of EHR databases, there will be more and better data available in the future.

The proposed DPM is built under the framework of landmarking, which does not require strong assumptions regarding the data generating mechanism. However, the approach still needs a working model, this being a spline-based Cox model in this study, and the working model may be mis-specified to a degree at some landmarks. This drawback is shared by other landmark models [36]. From a practical perspective, the working model in the landmarking approach can be flexibly and carefully specified after gathering information about the study hypotheses and population. In a complicated problem, dynamic prediction using the landmarking approach is still feasible and useful, but implementation may be difficult for the joint modeling approach, which needs strong assumptions on the data generating process.

A model-based calibration method was applied in this study because it could be easily extended to the Cox model. It may be valuable to use some other calibration methods and compare them in the future. The results in this study show a good calibration of the DPMs. It is believable that when the proposed DPM is applied in other settings, such as the data from other countries or data collected in the future, the quality of model calibration may decline. This means that we should be careful about model generalization. Then, the DPM needs to be re-calibrated to be able to provide reliable predictions. Further study for calibrating the DPM as well as full external validation is needed.

A major strength of our study was the use of longitudinal lab values, ECOG PS, and relatively robust mortality data from the Flatiron Health nationwide real-world EHR database, which is broadly representative of advanced NSCLC patients in the US [17]. There were several limitations to this paper. Firstly, the proposed DPM used spline basis functions. In this paper, Wendland basis functions were used and the number of basis functions was determined according to the range of landmarks. The choice of basis function and number of nodes was decided by the authors’ preference. Second, the proposed DPM did not consider the dependence of measurement times on the patient’s clinical factors. As this paper is interested in short-term prediction, this problem may not influence the accuracy of prediction in a meaningful way. Third, we believe including longitudinal treatment information may improve long-term survival. However, along with the disease course, there will be more missingness on the longitudinal predictors, which could reduce the prediction accuracy of DPMs. In summary, the proposed DPM may be practically valuable in improving end-of-life care.

## 5. Conclusions

In terms of their ability to discriminate poor and better prognosis NSCLC patients, the performance of prediction models can be substantially improved by incorporating time-varying patient features and associations that vary over the disease course, especially for near-term prediction. Remarkably, the DPM using time-dependent albumin may be as good as, or even better than, using time-dependent ECOG PS, which is desirable because albumin is an objective clinical measurement unlike ECOG PS. A DPM using both time-dependent albumin and ECOG PS performs slightly better than when using either alone or in combination with baseline features.

## Figures and Tables

**Table 1:** Summary of patient characteristics at baseline.

Patient Characteristic		Overall (*n* = 14,605)
Age at initiation of first-line therapy (year) (mean (SD))		68.2 (9.6)
Albumin at initiation of first-line therapy (g/dL) (mean (SD))		3.7 (0.5)
Lymphocyte at initiation of first-line therapy (109/L) (mean (SD))		1.4 (0.7)
Weight at initiation of first-line therapy (kg) (mean (SD))		74.9 (18.4)
Gender	Female	6769 (46.3%)
	Male	7836 (53.7%)
Histology *	NSCC	9917 (67.9%)
	NOS	671 (4.6%)
	SCC	4017 (27.5%)
Smoking status	History of smoking	13,071 (89.5%)
	No history of smoking	1534 (10.5%)
Race/ethnicity	White/non-Hispanic	10,498 (71.9%)
	Asian/non-Hispanic	295 (2.0%)
	Black/non-Hispanic	1197 (8.2%)
	Hispanic	340 (2.3%)
	Other	2275 (15.6%)
Targetable mutation	Mutated type	1228 (8.4%)
	Wild type/undocumented	13,377 (91.6%)
ECOG PS at initiation of first-line therapy	<2	11,563 (79.2%)
	≥2	3042 (20.8%)
First-line treatment group	Chemotherapy	7436 (50.9%)
	Chemotherapy + monoclonal antibody **	1831 (12.5%)
	Chemoimmunotherapy	1734 (11.9%)
	Immunotherapy	1999 (13.7%)
	Tyrosine kinase inhibitor	1031 (7.1%)
	Any study	473 (3.2%)
	Other	101 (0.7%)

* NSCC—non-squamous lung cancer, SCC—squamous cell lung cancer, NOS—not otherwise specified. ** About 97% of the monoclonal antibody was bevacizumab, and others included necitumumab, ramucirumab, trastuzumab, etc.

## Data Availability

Restrictions apply to the availability of these data. The data that support the findings of this study have been originated by Flatiron Health, Inc. These de-identified data may be made available upon request, and are subject to a license agreement with Flatiron Health; interested researchers should contact <DataAccess@flatiron.com> to determine licensing terms.
